# Efficacy and Safety of GLP-1 Receptor Agonists in Patients With Type 2 Diabetes Mellitus and Non-Alcoholic Fatty Liver Disease: A Systematic Review and Meta-Analysis

**DOI:** 10.3389/fendo.2021.769069

**Published:** 2021-12-09

**Authors:** Yuan Zhu, Jiao Xu, Dong Zhang, Xingyu Mu, Yi Shi, Shangtao Chen, Zengxiang Wu, Shuangqing Li

**Affiliations:** Department of General Practice, West China Hospital of Sichuan University, Chengdu, China

**Keywords:** intrahepatic adipose, hepatic fibrosis, GLP-1 receptor agonists, non-alcoholic fatty liver disease, type 2 diabetes, meta-analysis

## Abstract

The prevalence of non-alcoholic fatty liver disease (NAFLD) in patients with type 2 diabetes mellitus (T2DM) is increasing and there is an urgent need for new treatment strategy to prevent progression of hepatic steatosis and fibrosis. We have performed a systematic review and meta-analysis of randomized controlled trials (RCTs) to evaluate the efficacy and safety of glucagon-like peptide-1 receptor agonists (GLP-1RAs) in the treatment of hepatic steatosis and fibrosis in patients with T2DM and NAFLD. The PubMed, Web of Science, Scopus, Embase and Cochrane Central Register of Controlled Trials databases were searched for articles that met the eligibility criteria to explore the efficacy and safety of GLP-1RAs in patients with T2DM and NAFLD. We assessed pooled data using a random/fixed-effects model according to the *I^2^* and p-values. Eight trials that included a total of 468 participants were eligible for inclusion in the review. For primary outcomes, administration of GLP-1RAs significantly decreased the content of intrahepatic adipose (IHA)[p=0.007, weight mean difference (WMD) -3.01, 95% confidence interval (CI) -4.75, -1.28], subcutaneous adipose tissue (SAT) (p<0.00001,WMD -28.53,95%CI -68.09,-26.31), and visceral adipose tissue (VAT) (p<0.0001,WMD -29.05,95%CI -42.90,-15.9). For secondary outcomes, GLP-1RAs produced a significant decrease in levels of alanine aminotransferase(ALT)(p=0.02, WMD -3.82, 95%CI -7.04, -0.60), aspartate aminotransferase (AST) (p=0.03, WMD -2.4, 95%CI -4.55,-0.25, *I^2^ = *49%), body weight (p<0.00001,WMD -3.48,95%CI -4.58,-2.37), body mass index (p<0.00001,WMD -1.07,95%CI -1.35,-0.78), circumference waist (p=0.0002,WMD -3.87, 95%CI -5.88, -1.86) fasting blood glucose (p=0.02, WMD -0.35, 95%CI -0.06, -0.05), HbA_1c_ (p<0.00001,WMD -0.39,95%CI -0.56,-0.22), HoMA-IR(p=0.005, WMD-1.51, 95%CI-0.87,-0.16), total cholesterol (p=0.0008, WMD -0.31, 95%CI -0.48, 0.13) and triglycerides (p=0.0008, WMD -0.27, 95%CI -0.43,-0.11) in comparison with the control regimens. The main adverse events associated with GLP-1RAs included mild-to-moderate gastrointestinal discomfort and nonsense hypoglycemia that resolved within a few weeks. GLP-1RAs were an effective treatment that improved intrahepatic visceral and subcutaneous adipose tissue, inflammatory markers, the anthropometric profiles and some metabolic indices in patients with T2DM and NAFLD, GLP-1RAs could be considered for use in these if there are no contraindications. Further studies are needed to understand the direct and indirect effects of GLP-1RAs on NAFLD and the potential mechanism *via* which they prevent its progression.

**Systematic Review Registration**: PROSPERO, identifier CRD42021265806.

## 1 Introduction

The rising incidence of type 2 diabetes mellitus (T2DM) is a major concern in health care worldwide. According to global epidemiological data, approximately 462 million individuals were confirmed to have T2DM in 2017 (1). Non-alcoholic fatty liver (NAFLD) and T2DM commonly coexist in clinical practice. Epidemiological evidence suggests a strong bidirectional relationship between T2DM and NAFLD (2). Approximately 50%-70% of person with diabetes have NAFLD (3). NAFLD is a strong clinical signal for insulin resistance and metabolic syndrome and is considered to be a confirmative risk factor for T2DM (4). According to a meta-analysis of 80 trials from twenty countries (5), the global prevalence rates of NAFLD, non-alcoholic steatohepatitis (NASH), and advanced fibrosis in patients with T2DM were 55.5%, 37.3% and 17.0%, respectively. The existence of T2DM is closely associated not only with advanced fibrosis in cross-sectional data (6, 7), but also with rapid progression of hepatic fibrosis (8, 9). In Japan, liver-related disease is the third leading cause of mortality (9.3%) in patients with T2DM (10).

NAFLD is a major cause of liver disease worldwide. In US, NASH has become the leading cause of end-stage liver disease and the main indication for liver transplantation. The major risk of NAFLD include alcohol consumption, obesity, T2DM, and metabolic syndrome. It has become increasingly clear that NAFLD is the hepatic manifestation of metabolic syndrome and is highly prevalent in obese and diabetic subjects (11). NAFLD encompasses a broad clinical spectrum ranging from non-alcoholic fatty liver to NASH, advanced fibrosis, cirrhosis, and hepatocellular carcinoma (12). NASH can be called “diabetic hepatopathy”. A meta-analysis of epidemiology studies suggested that NAFLD has a global prevalence of 25.24%, with the highest prevalence rates in the Middle East and South America and the lowest rate in Africa (13). NASH has been calculated to be present in 2%-5% of the general population (8), while NAFLD accounts for approximately 20% of cases of NASH (6). Until now, liver biopsy has been the gold standard for identifying simple steatosis, NASH, or fibrosis. From a histological point of view, only more than 5% of hepatocytes undergo degeneration called simple steatosis. Diagnosis of NASH necessary requires steatosis (more than 5%), and both lobular inflammation and ballooning degeneration of hepatocytes with a mainly zone 3 distributions (14). NASH gradually progresses to fibrosis, cirrhosis, and hepatocellular carcinoma. Hepatic fibrosis shows intrahepatic connective hyperplasia and deposition. There are 4 stages according to severity, stage1 (perivenular, perisinusoidal, or periportal fibrosis), stage 2 (both zone 3 and periportal fibrosis), stage 3 (bridging fibrosis), and stage 4 (cirrhosis) (15).

The pathogenesis of NAFLD involves a complex interaction between environmental factors, obesity, changes in the microbiota, and predisposing genetic variants and results in a disturbed lipid homeostasis and an excessive accumulation of triglycerides and other lipid species in hepatocytes (16, 17). Lipotoxicity and inflammation are the main pathogenic factors associated with NASH (18, 19). Moreover, there is growing evidence that overnutrition negatively interfere with immune system. In the progression of NAFLD, there is a specific link between fat accumulation and inflammation. Adipokines (the most abundant adipokines included leptin and adiponectin) produced by white fat are key factors between metabolism and immunity. At the same time, the two adipokines have different roles in inducing inflammatory response. Leptin upregulates TNF-a and IL-6, and is associated with insulin resistance and T2DM. In contrast, adiponectin has anti-inflammatory properties and downregulates the expression and release of proinflammatory immune mediators, for example, κB activation and TNF-α expression (20, 21). Besides, adiponectin anti-aliphatic activity in hepatocytes through increasing oxidation of free fatty acids and decreasing gluconeogenesis, free fatty acid flow and *de novo* lipogenesis (22). It has evidence support that serum leptin levels increased in NAFLD patients, while serum adiponectin levels decreased (23), which is mainly related to the characteristics of two kinds of adipokines. In addition, systemic insulin resistance to progression of non-alcoholic fatty is the main driver of nonalcoholic fatty liver hepatitis, cirrhosis, and liver cancer (17, 18). NAFLD and NASH impose a substantial economic burden and are responsible for poor health-related quality of life (11, 24).

As we all know, liver biopsy is the gold standard for diagnosing NAFLD. However, it has well-known limitations, including invasiveness, poor acceptability, sampling variability, and cost. As a result, noninvasive strategies are gradually replacing liver biopsy diagnosis NAFLD. These strategies including a “biological” approach based on the quantification of biomarkers in serum samples or a “physical” approach based on the measurement of liver stiffness, using either ultrasound or magnetic resonance-based elastography and so on techniques. However, in previous researches suggested that there was no significant correlation between serum biomarkers level, for example liver enzymes, and NAFLD (25, 26). In addition, patients with advanced liver disease show decreased alanine aminotransferase (ALT) levels. Thus, it is very unreasonable for us to diagnosis NAFLD only rely on the levels of serum biomarkers. It is important that we should combine serum biomarkers and physical measurements. We know that Fibroscan allows a rapid assessment and is reasonably accurate for diagnosing the presence of steatosis (27). However, the controlled attenuation parameter (CAP), measured by Fibroscan, is limited by high failure rates in obesity, lack of exact anatomic localization, and low accuracy for quantifying the amount of steatosis. Compared with MRI, studies found that the detection sensitivity is poor for patients with low fat concentrations using computerized tomography (CT) (28, 29). Magnetic Resonance Imaging (MRI) can detect small amounts of liver fat and is considered the most accurate non-invasive method to quantify liver fat. MRI proton density fat fraction (MRI-PDFF) corrects for imaging confounders that can affect liver fat measurement and has emerged as an accurate, reproducible biomarker of liver fat (30, 31). In a longitudinal assessment, MRI-PDFF was more accurate at detecting changes in liver fat than liver biopsy (32, 33). MRI, as noninvasive approach, diagnosis of NAFLD is very promising in future.

GLP-1 receptor agonists (GLP-1RAs) are gut-derived incretin hormone that induces weight loss and insulin sensitivity. These agents act directly on human hepatocytes *in vitro*, reducing steatosis by decreasing *de-novo* lipogenesis and increasing fatty acid oxidation (34, 35). In the LEAN trial, which investigated the effects of 48 weeks of treatment with liraglutide 1.8 mg on liver histology, resolution of NASH without worsening of fibrosis was found in nine of 23 patients randomized to liraglutide but in only two of 22 patients randomized to placebo (36).

Furthermore, several randomized controlled trials (RCTs) have compared the efficacy and safety of GLP-1RAs with that of other active agents or placebo in patients with T2DM and NAFLD. However, the sample sizes were small in all of these studies. Therefore, comprehensive evaluation of the various studies requires a meta-analysis. We hypothesized that more significant improvement of hepatic fibrosis and steatosis may be achieved by GLP-1RAs than by other antidiabetic agents or placebo in patients with T2DM and T2DM. This systematic review and meta-analysis were performed to summarize current evidence for the efficacy and safety of GLP-1RAs in these patients.

## 2 Material and Method

### 2.1 Protocol

This research was conducted in accordance to the Preferred Reporting Items for Systematic Reviews and Meta-analyses (PRISMA) guidelines. The protocol for the review was registered with PROSPERO (number CRD42021265806).

### 2.2 Search Strategy

Our search strategy composed both entry terms and MeSH terms. We searched the PubMed, Web of Science, Scopus, Embase, and Cochrane Central Register of Controlled Trials databases for relevant articles published through to July 10, 2021 using the following combination of key terms: GLP-1RAs, liraglutide, exenatide, dulaglutide, T2DM, diabetes mellitus type 2, type 2 diabetes mellitus, non-alcoholic fatty liver disease, NAFLD, liver, non-alcoholic fatty, and RCTs. The details of our search strategy are provided in the **Supplementary Material 1**. No language restrictions were imposed.

### 2.3 Study Selection

Studies that met the following inclusion criteria were selected for review: 1) study population comprised of participants with a definitive diagnosis of T2DM and NAFLD; 2) participants aged >18 years; 3) a study period of at least 12 weeks; 4) GLP-1 RAs used in the intervention group; 5) inclusion of a control group; 6) documentation of intrahepatic adipose (IHA), visceral adipose tissue (VAT), and subcutaneous adipose tissue (SAT) on ultrasonography/CT/MRI scans and inflammatory marker levels; and7) SAT, VAT and gamma-glutamyl transpeptidase (GGT) levels included as outcomes. The following exclusion criteria were applied: 1) Articles published as editorials, letters, reviews, brief reports or book chapters, along with non-randomized and observational studies; 2) a single study of NAFLD or NASH; 3) inclusion of patients with secondary hepatic disease. Two reviewers (YZ, JX) independently screened titles and abstracts and then independently screened the full-text articles. Any disagreements between the two reviewers were resolved by discussion.

### 2.4 Data Extraction and Risk of Bias Appraisal

Two reviewers (YZ, JX) independently extracted the following information from eligible studies: authorship, publication year, age, sex, body mass index (BMI), waist circumference, blinding method, study arms, sample size, dosage and duration of treatment and the strategy used to evaluate the primary outcome. We also extracted data for IHA, SAT, VAT from MRI scans, data for inflammatory markers [ALT, aspartate aminotransferase (AST) and GGT], hepatic fibrosis, anthropometric profiles and metabolic indices before and after treatment. Two authors (YZ, JX) independently assessed the quality of the included RCTs according to the Cochrane Collaboration Tool guidelines, which contained the following seven domains: random sequence generation (selection bias); allocation concealment (selection bias); blinding of participants and personnel (performance bias); blinding of outcome assessment (detection bias); incomplete outcome data (attrition bias); selective reporting (reporting bias), and other bias. Any disagreement was discussed between the two researchers or resolved by a third researcher (SL).

### 2.5 Statistical Analysis

All the variables analyzed in this review and meta-analysis were continuous and are presented as the mean and standard deviation (SD). When a discrete trend in a study was represented by the standard error, we converted this to the SD using a standard formula. The weighted mean difference (WMD) and 95% confidence interval (CI) were used. All statistical analyses were performed using RevMan version 5.4 software (Cochrane Collaboration, Oxford, UK).

Study heterogeneity was tested using the *I^2^* statistic, which is a quantitative measure of inconsistency across studies. In general, studies with an *I^2^* statistic of 0%–50% are considered to have low statistical heterogeneity, and we used RevMan with a fixed-effects model to pool continuous variables. However, studies with an *I^2^* statistic >50% were considered to have high heterogeneity, and it was necessary to use a random-effects model and the heterogeneity test in RevMan.

## 3 Results

### 3.1 Search Process

**Figure 1:**
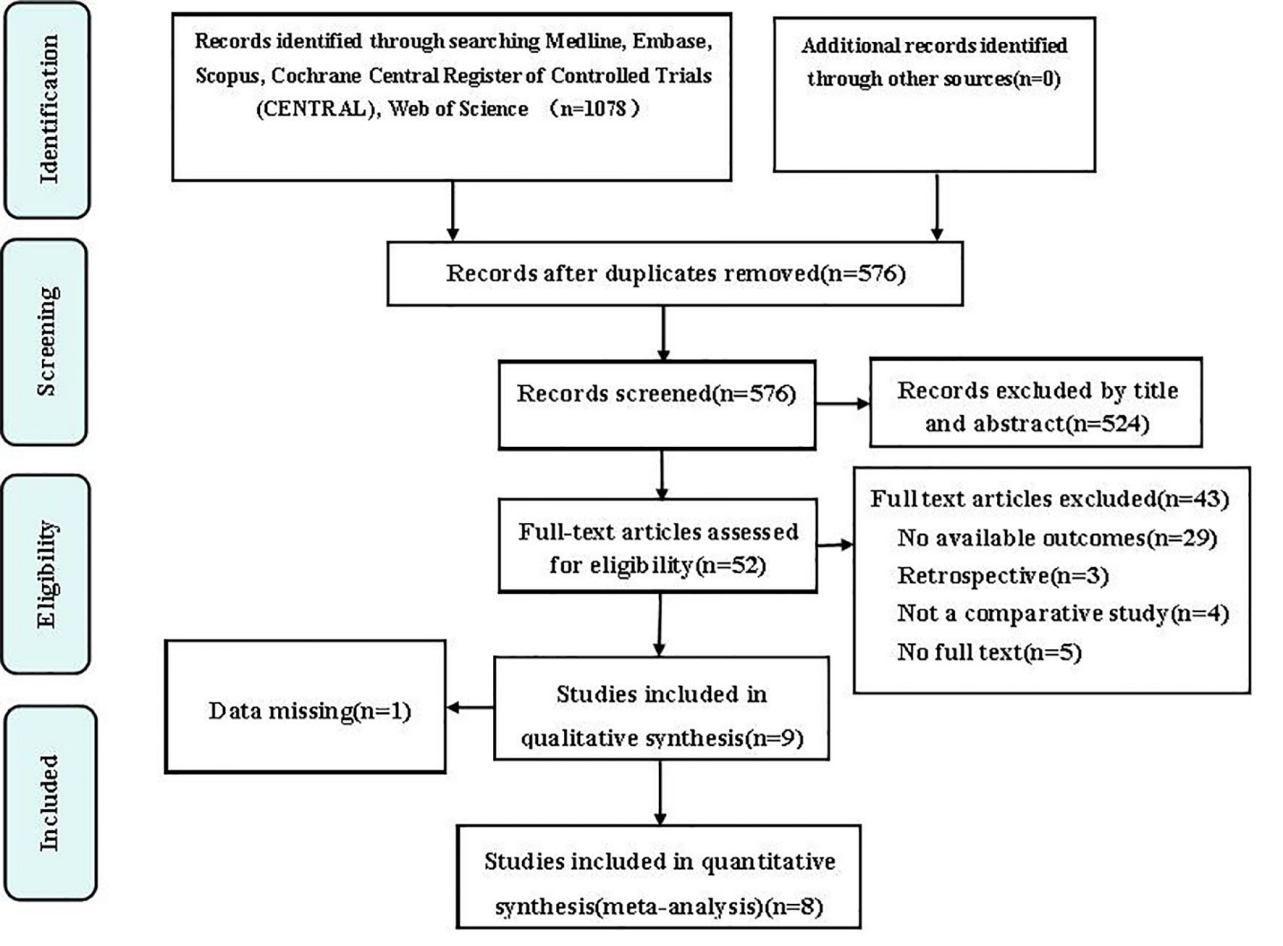
Flow chart showing the study identification and selection process.

The initial search of the electronic databases identified a total of 1078 studies, 502 of which were removed for being duplicates. Fifty-two records were selected for further assessment after screening of their titles and abstracts, 43 of which were excluded for the following reasons: no available outcomes data (n=29), retrospective study design (n=3); no comparator (n=4), no full text version available (n=5); and a limited follow-up duration (n=2). Nine full-text RCTs were included in the qualitative analysis. One of these studies was subsequently found to have missing data and was excluded, leaving eight eligible articles (37–44), that included 12 interventional or control groups for inclusion in the meta-analysis. **Figure 1** shows the study identification and selection process.

### 3.2 Study Characteristics

The detailed characteristics of the eight RCTs included in our meta-analysis are listed in **Table 1**. All the studies (37–44) were published between 2014 and 2020, included a total of 468 patients, and had sample sizes of 22–71 (median, 50). The follow-up duration was 12 weeks. Five studies (38, 41–44) of GLP-1RAs used liraglutide, two studies (37, 40) used exenatide and one studies (39) used dulaglutide to treat patients with T2DM and NAFLD. Two studies compared liraglutide vs placebo, two compared liraglutide vs sitagliptin, two compared liraglutide vs insulin, and two studies compared exenatide vs insulin. Proton nuclear magnetic resonance spectroscopy was used to examine the intrahepatic fat content (37, 38, 40, 41, 43). The liver proton density fat fraction was measured using magnetic resonance spectroscopy in three trials (39, 42, 44). In most cases, changes in liver fat content before and after treatment were documented by MRI scans.

**Table 1:** T he main characteristics of the included studies.

Author/Year	Arms	Blinding	Body mass index		Waist circumference		Male		Sample(N)		Intervention (Dose)		Study duration	Outcome evaluation
			GLP-1 RAs	Control	GLP-1 RAs	Control	GLP-1 RAs	Control	GLP-1 RAs	Control	GLP-1 RAs	Control group		
Guo et al./2020 (38)	3	No	29.2± 4.2	28.6± 3.7	95.5± 8.0	98.6± 7.3	16	20	31	30	Liraglutide(max 1.8mg qd)	Placebo	26	^1^H-MRS
Guo et al./2020 (38)	3	No	29.2± 4.2	28.3± 3.8	95.5± 8.0	96.3± 7.6	16	18	31	30	Liraglutide(max 1.8mg qd)	Insulin	26	^1^H-MRS
Kuchay et al./2020 (39)	2	Yes	29.6± 3.6	29.9± 3.9	_	_	13	12	32	32	Dulaglutide(max 1.5mg qw)	Usual care	24	MRI-PDFF
Liu et al./2020 (40)	2	No	28.49 ± 3.02	27.84 ± 3.10	97.17 ± 9.35	97.33± 9.20	16	17	35	36	Exenatide(19.43 ± 2.36ug qd)	Insulin(16.97 ± 8.83 IU)	24	MRS
Zhang et al./2020 (43)	2	Yes	27.6± 5.2	27.1± 3.8	93.2 ± 4.6	91.6± 7.2	13	15	30	30	Liraglutide(max 1.2mg qd)	Pioglitazone(max 30mg qd)	24	^1^H-MRS
Yan et.al/2019 (42)	3	No	30.1± 3.3	29.6± 3.5	101.7 ± 7.9	102.9± 9.9	17	14	24	24	Liraglutide(max 1.8mg qd	Insulin	26	MRI-PDFF
Yan-1 et.al/2019 (42)	3	No	30.1± 3.3	29.7± 2.8	101.7 ± 7.9	102.8± 8.3	17	21	24	27	Liraglutide(max 1.8mg qd	Sitagliptin(100mg qd)	26	MRI-PDFF
Smits et.al/2016 (41)	3	Yes	32.8± 1.0	30.6± 0.7	_	_	8	9	17	17	Liraglutide(max 1.8mg qd)	Placebo	12	^1^H-MRS
Smits-1 et.al/2016 (41)	3	Yes	32.8± 1.0	32.8± 1.0	_	_	8	10	17	17	Liraglutide(max 1.8mg qd)	Sitagliptin(100mg qd)	12	^1^H-MRS
Tang et.al/2015 (44)	2	Yes	31.3 ± 6 4.1	31.2 ± 6 5.0	107.0± 10.6	105.4± 11.0	11	11	18	17	Liraglutide(max 1.8mg qd)	Insulin	12	MRI+MRI-PDFF
Bi et al./2014 (37)	3	No	25.1± 1.1	24.5± 0.6	93.2± 2.5	89.5± 1.9	7	4	11	11	Exenatide(max 10ug bid)	Insulin	24	^1^H-MRS
Bi et al./2014 (37)	3	No	25.1± 1.1	23.9± 1.0	93.2± 2.5	87.3± 2.4	7	5	11	11	Exenatide(max 10ug bid)	Pioglitazone(max 45mg qd)	24	^1^H-MRS

GLP-1 RAs, Glucagon Like Peptide-1 receptor agonists; MRI, magnetic resonance imaging; ^1^H-MRS, magnetic resonance spectroscopy; MRI-PDFF proton density fat fraction was measured by MRS.

### 3.3 Quality Assessment

**Figure 2:**
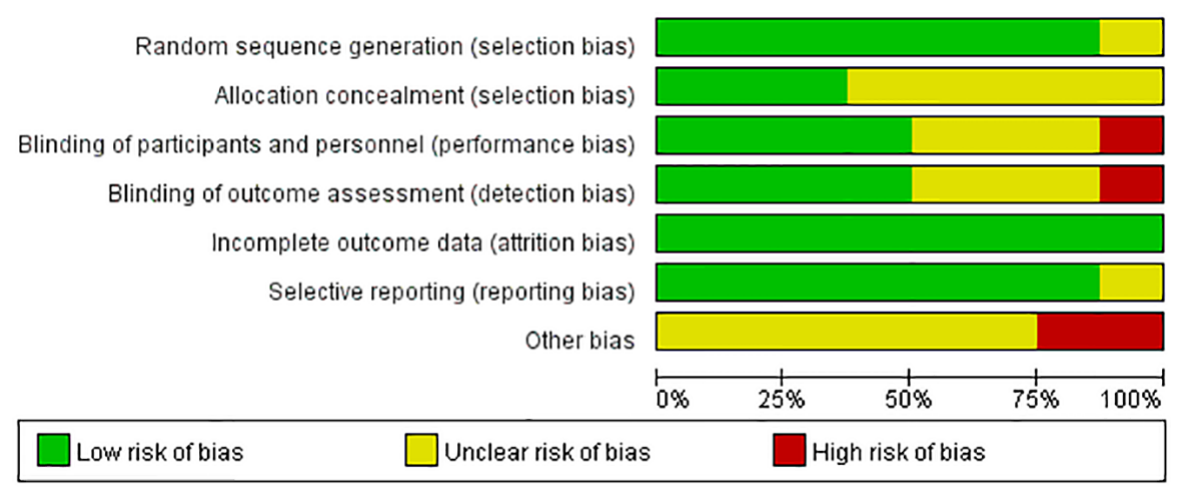
Risk of bias graph: Authors’ judgements about each risk of bias item are presented as percentages across all included studies.

**Figure 3:**
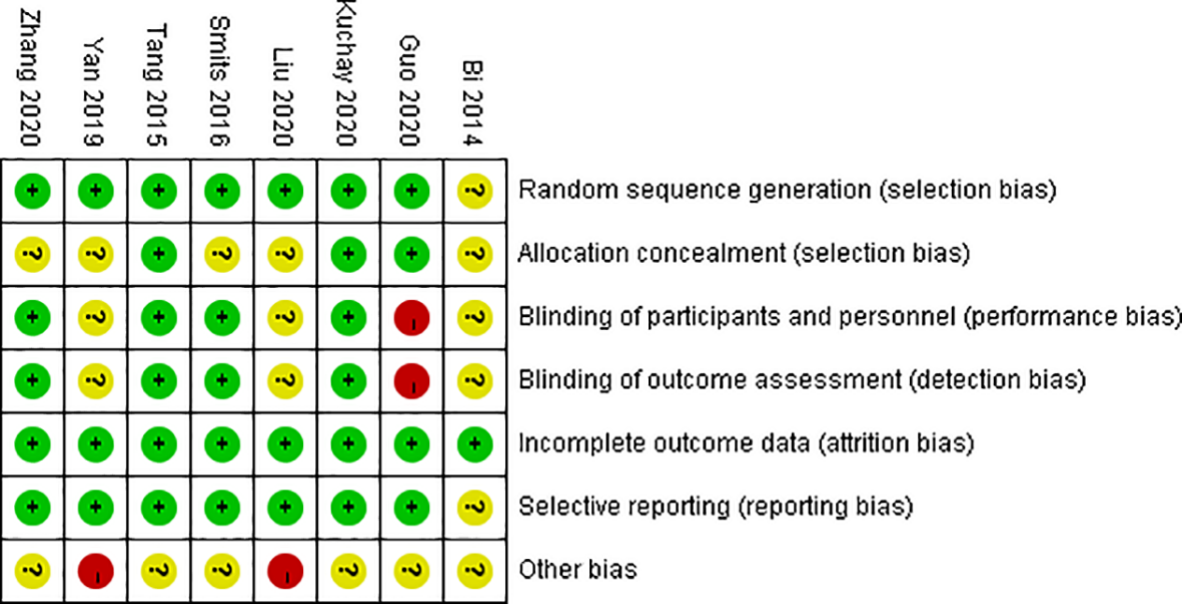
Risk of bias summary: Authors’ judgements about each risk of bias item for each included study.

We evaluated the risk of bias in the included studies using the Cochrane Risk of Bias Tools. We scored each domain as “low”, “high” or “unclear” risk of bias. The domains that were not explicitly proposed in this study were expressed as uncertain. Seven of the eight trials included in the final analysis included details of the randomization sequence generation method used, such as computerized randomization sequence and a random number table. Only two studies used allocation concealment. Most of the studies did not describe the allocation concealment method used in their research, so we expressed it as unclear. One trial clearly pointed out its lack of blinding. Two trials used intention-to-treat analysis, and in the other six studies the loss to follow-up rates in subjects in the GLP-1 RA groups and control groups were very similar. Two trials acknowledged that their research had other bias, the primary cause of which was their open-label design. Based on the above limitations, we included two studies of good quality, two of moderate quality, and four of poor quality, the details are shown in **Figures 2** and **3**.

### 3.4 Outcomes

The primary outcome of our meta-analysis of the efficiency of GLP-1 RAs in patients with T2DM and NAFLD, included IHA, SAT, VAT, and Fibrosis-4 (FIB-4) index and the NAFLD fibrosis score (NFS). The secondary outcomes included hepatic enzyme levels (ALT, AST, GGT), anthropometric variables (body weight, BMI and waist circumference), and metabolic indices (glycemic indices, serum lipid levels, and blood pressure).

#### 3.4.1 Effect on Intrahepatic Adipose

**Figure 4:**
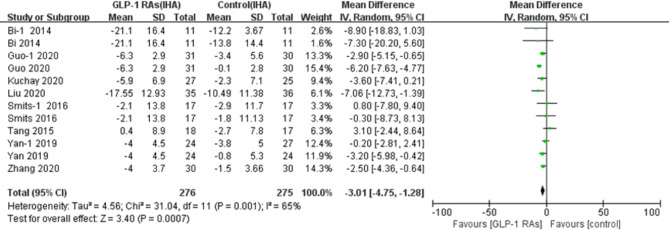
Glucagon-like peptide-1 receptor agonists (GLP-IRAS); intrahepatic adipose (IHA); SD, standard deviation; CI, confidence interval; IV, inverse variance; Bi-l, concluded exenatide vs pioglitazone; Guo-l, concluded liraglutide vs insulin; Smits-l, concluded liraglutide vs placebo; Yan-1, concluded liraglutide vs sitagliptin.

The studies that included investigation intrahepatic fat content included 468 participants. Meta-analysis revealed that GLP-1 RA regimen had a significant effect on the hepatic adipose content (p=0.007, WMD -3.01, 95%CI -4.75, -1.28, *I^2^ = *65%, **Figure 4**) in comparison with controls, with statistically significant between-study heterogeneity.

#### 3.4.2 Effect on Subcutaneous And Visceral Fat

**Figure 5:**
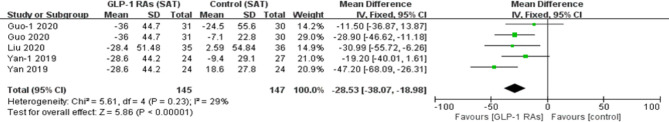
Glucagon-like peptide-1 receptor agonists (GLP-lRAs); subcutaneous fat (SAT); SD, standard deviation; CI, confidence interval; IV, inverse variance; Guo-l, concluded liraglutide vs insulin; Yan-1, concluded liraglulide vs sitagliptin.

**Figure 6:**
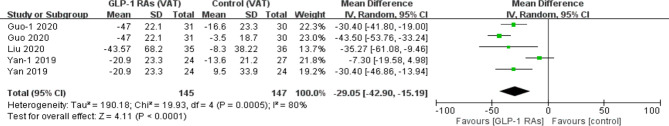
Glucagon-like peptide-1 receptor agonists (GLP-lRAs); visceral fat (VAT); SD, standard deviation; CI, confidence interval; IV, inverse variance; Guo-l, concluded liraglutide vs insulin; Yan-1, concluded liraglutide vs sitagliptin.

We conducted a meta-analysis of SAT and VAT in three trials that included 237 patients and found that GLP-1 RAs significantly changed the SAT (p<0.00001, WMD=-28.53, 95%CI -68.09, -26.31, *I^2^ = *29%) and VAT (p<0.0001, WMD -29.05, 95%CI -42.90, -15.9, *I^2^ = *80%, **Figures 5** and **6**). Pooled analysis of the three studies revealed statistically significant between-study heterogeneity in VAT but not in SAT.

#### 3.4.3 Effect on Hepatic Fibrosis

**Figure 7:**
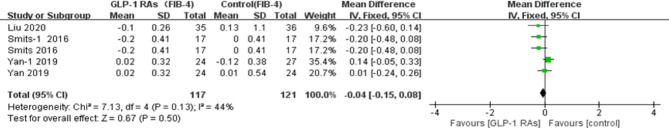
Glucagon-like peptide-1 receptor agonists (GLP-1RAs); Fibrosis-4 index (FIB-4); SD, standard deviation; CI, confidence interval; IV, inverse variance; Smits-l, concluded liraglutide vs placebo; Yan-1 , concluded liraglutide vs sitagliptin.

**Figure 8:**
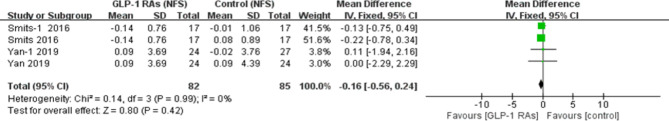
Glucagon-like peptide-1 receptor agonists (GLP-1RAs); NAFLD fibrosis score (NHS); SD, standard deviation; CI, confidence interval; IV, inverse variance; Smits-l, concluded liraglutide vs placebo; Yan-1, concluded liraglutide vs sitagliptin.

Three trials (40–42) in the meta-analysis included measurement of the FIB-4 index. One study (42) reported that there was no significant change in the value of FIB-4 index between baseline and treatment with an GLP-1RA. Meta-analysis did not find any significant effect of treatment with an GLP-1RA on the FIB-4 index value (p=0.50, WMD -0.04, 95%CI -0.15, 0.08, *I^2^ = *44%, **Figure 7**), with no significant between-study heterogeneity. Only two studies (41, 42) assessed changes in NFS after treatment with a GLP-1RA. Similarly, there was no significant between-group difference in the NFS value (p=0.42, WMD -0.16, 95%CI -0.56, 0.24, *I^2^ = *0%, **Figure 8**) or any significant between-study heterogeneity.

#### 3.4.4 Effect on Hepatic Enzyme Levels

**Figure 9:**
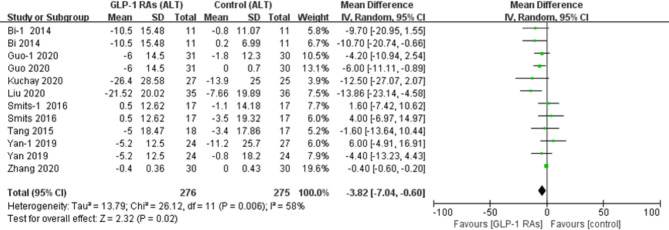
Glucagon-like peptide-1 receptor agonists (GLP-lRAs); alanine aminotransferase (ALT); SD, standard deviation; CI, confidence interval; IV, inverse variance; Bi-l, concluded exenatide vs pioglitazone; Guo-l, concluded liraglutide vs insulin; Smits-l, concluded liraglutide vs placebo; Yan-1, concluded liraglutide vs sitagliptin.

**Figure 10:**
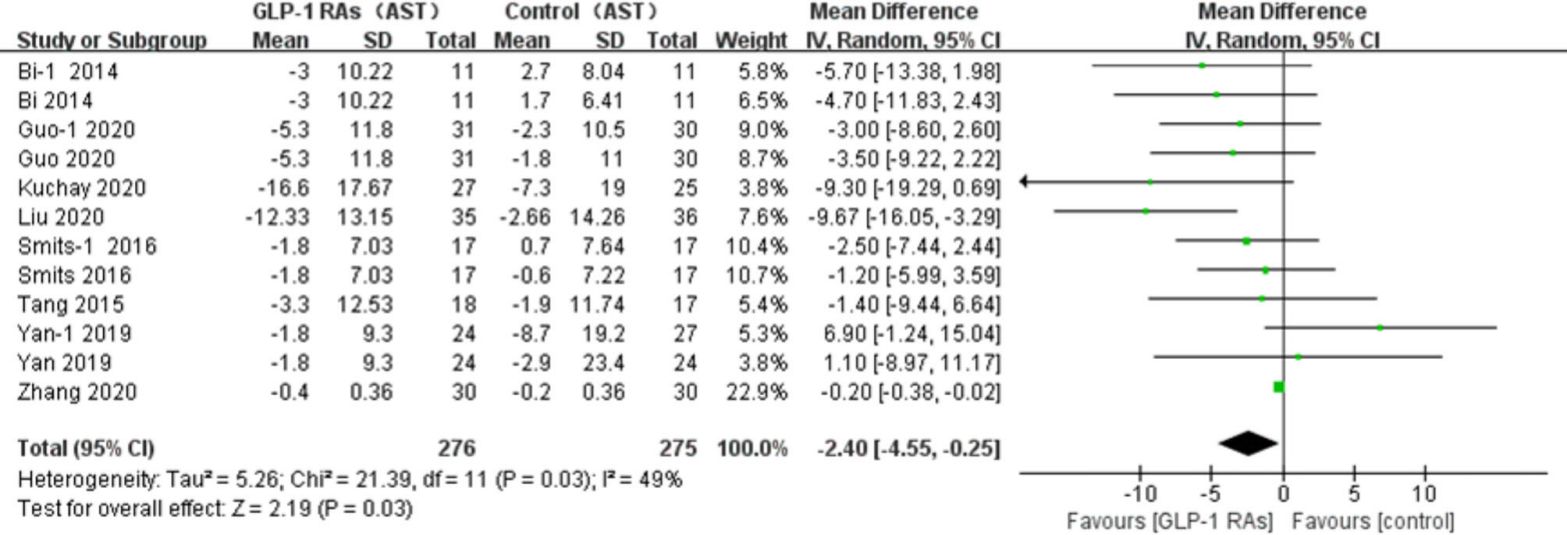
Glucagon-like peptide-1 receptor agonists (GLP-1RAs); aspartate aminotransferase (AST); SD, standard deviation; CI, confidence mterval; IV, inverse variance; Guo-l, concluded liraglutide vs insulin; Smits-l, concluded liraglutide vs placebo; Yan-1, concluded liraglutide vs sitagliptm.

**Figure 11:**
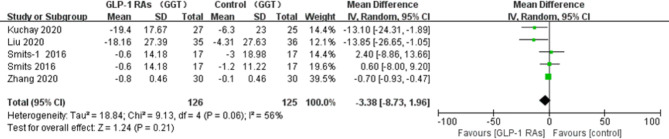
Glucagon-like peptide-I receptor agonists (GLP- 1 RAs); gamma-glutamyl transpcptidase (GGT); SD, standard deviation; CI, confidence interval; IV, inverse variance; Smits-l, concluded liraglutide vs placebo.

All eligible RCTs included ALT and AST as outcome indices. Notably, compared with controls, there was a significant decrease in the ALT level (p=0.02, WMD -3.82, 95%CI -7.04, -0.60, *I^2^ = *58%, **Figure 9**), and AST level (p=0.03, WMD -2.4, 95% CI -4.55, -0.25, *I^2^ = *49%, **Figure 10**) after treatment with a GLP-1RA. Similarly, we identified three trials with a total of 217 participants that included the GGT level. We found that treatment with a GLP-1RA had no significant effect on GGT (p=0.21, WMD -3.38, 95%CI -8.73, 1.96, *I^2^ = *56%, **Figure 11**). However, the meta-analysis found statistically significant between-study heterogeneity in liver enzyme levels.

#### 3.4.5 Effect on Anthropometric Variables

Overall, there was a significant reduction in body weight (p<0.00001, WMD -3.48, 95%CI -4.58, -2.37, *I^2^ = *55%) and BMI (p <0.00001, WMD -1.07, 95%CI -1.35, -0.78, *I^2^ = *35%) fowling treatment with a GLP-1RA. Six studies (37, 38, 40, 42–44) reported waist circumference data. Treatment with a GLP-1RA resulted in a statistically significant mean reduction in waist circumference compared with placebo or an active comparator (p=0.0002, WMD -3.87, 95%CI -5.88, -1.86, *I^2^ = *77%). However, there was statistically significant between-study heterogeneity (**Appendix 2**).

#### 3.4.6 Effect on Glycemic Indices

Treatment with a GLP-1RA produced statistically significant reductions in fasting blood glucose (FBG) (p=0.02, WMD -0.35, 95%CI -0.06, -0.05, *I^2^ = *13%), HbA_1c_ (p <0.00001, WMD -0.39, 95%CI -0.56, -0.22, *I^2^ = *5%) and homeostasis model assessment of insulin resistance (HoMA-IR) (p=0.005, WMD -1.51, 95%CI -0.87, -0.16, *I^2^ = *92%) in compassion with the control group. However, there was high heterogeneity in HoMA-IR between four trials (37, 38, 42, 43). In contrast, there was no statistically significant change in postprandial blood sugar (PBG) (p=0.32, WMD -0.67, 95%CI -1.99, 0.64, *I^2^ = *64%) in comparison with other treatments, with statistically significant between-study heterogeneity (**Appendix 2**).

#### 3.4.7 Effect on Serum Lipid Levels

Treatment with a GLP-1 RA produced a significant change in serum total cholesterol (TC) (p=0.0008, WMD -0.31, 95%CI -0.48, -0.13, *I^2^ = *22%) and triglycerides (TG) (p=0.0008, WMD -0.27, 95%CI -0.43, -0.11, *I^2^ = *0%) in comparison with controls. However, GLP-1RAs did not have any observable effect on other serum lipids, such as and low- density lipoprotein (LDL) (p=0.49, WMD -0.07, 95%C -0.26, 0.13, *I^2^ = *51%) and high-density lipoprotein (HDL) (p=0.28, WMD -0.03, 95%CI -0.08, 0.02, *I^2^ = *30%) **Appendix 2**).

#### 3.4.8 Effect on Blood Pressure

Four of the eight studies (including a total of 214 patients) included a meta-analysis of blood pressure (BP). Treatment with a GLP-1RA did not lead to a significant decrease in systolic blood pressure (SBP) (p=0.09, WMD -2.52, 95%CI -5.40, 0.36) and diastolic blood pressure (DBP) (p=0.19, WMD 1.51, 95%CI -0.77, 3.80). The results for SBP and DBP appeared to show slow heterogeneity (*I^2^ = *0%). Therefore, it is likely that GLP-1 RAs had no obvious antihypertensive effect in comparison with controls (**Appendix 2**).

### 3.5 Sensitivity Analysis

Studies with an I^2^ statistic of >50% were considered to have high heterogeneity. We performed a sensitivity analysis to test for heterogeneity. Each trial included in the analysis was removed to assess the sources of heterogeneity. The RCTs in the meta-analysis that included IHA showed statistically significant heterogeneity. We removed each article from the eight studies individually, after deleting the study by Guo et al. (38), the I^2^ value decreased from 65% to 23% and there were changes in the p-value (from 0.0007 to 0.0004), and the effect of IHA [from a WMD of -3.01, (95%CI -4.75, -1.28) to a WMD of -2.38, (95%CI -3.70, -1.07)]. However, we did not observe the same change in body weight, which was included in all eight studies in the meta-analysis. The reason for these changes may be the baseline of intrahepatic fat, which was higher in study by Guo et al. (38).

The same method was used to remove each of the four trials that analyzed postprandial blood sugar (37, 40, 42, 43) from the meta-analysis, after deleting the study by Bi et al. (37), the I^2^ value decreased from 64% to 47%, the p-value changed markedly (from 0.32 to 0.04) and there was a change in the effect of postprandial blood sugar [from a WMD of 0.67, (95%CI -1.99, 0.64) to a WMD of -1.15, (95%CI -2.24, -0.06)].We attributed these changes to changes in the PBG level, which did not decrease significantly in comparison with controls. However, when we removed each of the four trials that measured GGT, there were no obvious changes in the I^2^ value, p-value, WMD and 95%CI. Therefore, we believe that the heterogeneity in GGT levels between these studies was stable. Other outcomes, such as AST, SAT, fasting blood glucose, and HbA1_c_, showed low heterogeneity, therefore, we did not perform further tests.

### 3.6 Adverse Events

Four of the eight trials (38, 42–44) reported adverse events as outcomes. The most common adverse events associated with GLP-1RAs were gastrointestinal reactions and asymptomatic hypoglycemia. Gastrointestinal discomfort included mainly nausea, vomiting and diarrhea. Liraglutide was the GLP-1RA most likely to cause the above-mentioned adverse reactions, which tended to occur more frequently at higher doses. Those adverse reactions often occur within the first few weeks. Yan et al. (42) reported that headache occurred in only one patient (4.2%). Overall, there were no serious events related to GLP-1 RAs.

### 3.7 Publication Bias

The number of eligible trials for each outcome of interest was < 10, so no funnel plot was used to assess potential publication bias.

## 4 Discussion

We have performed a comprehensive meta-analysis of the most commonly prescribed GLP-1 RAs based on the largest pool of GLP-1 RA trials for patients with T2DM and NAFLD to date. Our results show that, compared with placebo or other active comparators, GLP-1 RAs can significantly improve IHA, SAT, VAT, ALT, AST, body weight, BMI, waist circumference, FBG, percent HbA_1C_, HoMA- IR, TC and TG. In contrast, use of a GLP-1 RA did not lead to statistically significant changes in FIB-4, NFS, PBG, LDL, HDL, SBP or DBP compared with controls. The major adverse events reported for GLP-1RAs were mild-to-moderate gastrointestinal discomfort and asymptomatic hypoglycemia, which resolved within a few weeks.

Improved insulin resistance and weight loss are the cornerstones of NAFLD treatment. Insulin resistance in NAFLD patients is associated with reduced adiponectin secretion by adipocytes, which is mainly manifested by adiponectin mediated signaling down-regulation of fatty acid β -oxidation (FAO), inhibition of glucose utilization and fatty acid synthesis. What matters is that in patients with NAFLD, mechanistic studies demonstrated that GLP-1RAs is associated with improvements in *de novo* lipogenesis, β-oxidation, and IR (systemic, adipose, and hepatic) (45, 46). In addition, GLP-1 RAs can influence IR through promotion of weight loss (delayed gastric emptying, appetite suppression). HoMA-IR is an indicator to evaluate insulin resistance, which runs through the whole process of occurrence and development of NAFLD. In our study we found that GLP1-RAs can improve HoMA- IR, which further prove that the treatment of NAFLD is benefit from GLP-1RAs. We know that the most serious risk of death from NAFLD is cardiovascular disease. We know that GLP-1 RAs have additional roles in reducing the risk of major adverse cardiovascular events and cardiovascular deaths in high-risk patients with T2DM, especially in the Asian population (47–49). T2DM and NAFLD often coexist, GLP-1RAs has the potential to reduce cardiovascular risk in NAFLD patients (48, 50). However, this benefit was only shown with liraglutide, injectable semaglutide and dulaglutide long-term use.

Previous studies have shown that GLP-1RAs act directly on human hepatocytes to decrease steatosis by preventing regeneration of fat and increasing oxidation of fatty acids and have shown that GLP-1RAs reduce IHA without insulin existed (34, 51, 52). These findings indicate that GLP-1RAs decrease IHA and have no relationship with weight loss. However, one study found that weight reduction alleviated intrahepatic fat content and aminotransferase levels (53). Moreover, a systematic review that included 23 RCTs of the effect of lifestyle interventions, such as diet, physical activity, and/or exercise, on the hepatic indicators of steatosis showed strong associations of reduction in IHA and liver aminotransferase levels with weight loss (54). Moreover, although weight loss in the range of approximately 5% -7% can decrease steatosis, a weight reduction of 8%-10% is needed to reverse steatohepatitis (55). In addition, reduction in NAFLD, resolution of steatohepatitis, and regression of fibrosis occurred in patients with a weight loss of ≥10% (56). Exercise and diet control appeared to have limited effects on NAFLD, given that 3-6% of patients achieved weight loss by long-term lifestyle interventions and less than 50% attained a weight reduction of 7%.

In recently years, the clinical role of GLP-1RAs has been explored in the treatment of patients with T2DM and NAFLD. A large number of clinical studies has shown that GLP-1RAs are better than placebo or other active comparators in their ability to achieve a significant decrease in IHA in these patients (37–43, 57–61). Surprisingly, in one study (32), IHA was not significantly reduced by administration of liraglutide for 12weeks, but was significantly decreased by insulin. However, Yan et al. (26, 30) demonstrated that liraglutide could significantly reduce intrahepatic fat content when compared with the effect produced by insulin 24/26 weeks of follow-up. The inconsistent findings of these studies may reflect differences in follow-up duration, the baseline characteristics of the study population, and other factors. Further clinical studies are needed to clarify the effects of liraglutide and insulin in patients with T2DM and NAFLD.

Several studies suggested that GLP-1RAs produce a significant change in IHA that correlates with changes in weight and HbA_1C_ in patients with T2DM and NAFLD (37, 58, 62). These studies divided patients into two subgroups according to whether weight loss was <5% or >5%. Eventually, they concluded that the reduction in IHA in patients who achieved marked weight loss was significantly greater than that in those with less weight loss. Furthermore, Feng et al. (58) reported patients with T2DM and NAFLD whose HbA_1c_ decreased by≥2.5% showed more significant reductions in IHA than those whose HbA_1c_ decreased by<2.5%. Patients with an HbA_1c <_6.5% had a markedly lower IHA content than those with HbA_1c_≥6.5%. These results are similar to our present findings of a significant decrease in the content of hepatic adipose, BMI, and HbA1C in patients who received a GLP-1RA in comparison with controls. Therefore, these findings suggest that GLP-1RAs are effective in improving fatty liver and are closely related to weight loss, but have limited effects. Further studies are needed to understand the role of a GLP-1 RAs in combination with weight loss in the treatment of T2DM and NAFLD.

Our study showed that GLP-1RAs significantly decreased SAT and VAT content compared with the control, which is consistent with the results of previous studies. Likewise, this research suggests that GLP-1RAs are able to achieve significant decreases in ALT, AST, weight, waist circumference, fasting blood glucose, HbA_1c_, HoMA-IR, triglycerides and total cholesterol, which is consistent with previous RCTs (63–67). We further demonstrated the efficacy of GLP-1 RAs in improving liver enzymes, anthropometric variables, and some metabolic indices. However, in this study we did not find statistically significant difference in GGT or PBG levels when compared with controls, which differs from previous studies (63, 66, 67). However, these inconsistent findings may reflect differences in the baseline characteristics of the study populations and follow-up duration between studies. Further studies are needed to clarify the effects of GLP1 RAs on GGT and postprandial blood sugar levels.

We know that NASH is the most severe phase of NAFLD, and the primary objective of treatment for NASH is to prevent the development of cirrhosis. It is surprising that GLP-1RAs had a resolution effect on NASH, besides reducing IHA, liver enzymes and improving metabolic index. In a small phase II clinical trial of 52 patients with biopsy-proven NASH, patients receiving subcutaneous injections of liraglutide (1.8 mg per day for 48 weeks) achieved resolution of NASH than those receiving placebo (36). Although this finding was impressive, liraglutide may not be convincing enough to study histological improvements in small samples. Subsequently, in a phase II trial, 320 patients with biopsy-proven NASH were randomized to receive daily subcutaneous semaglutide 0.4/2.4mg or placebo for 72 /48weeks, found that subcutaneous injection of 0.4/2.4mg of semaglutide had a resolution effect on biopsy-confirmed NASH, but no effect was found on fibrosis reversal (68) (NCT02970942/NCT03987451). Based on those results, semaglutide is expected to prove its benefit as a treatment for NASH through additional phase 3 clinical trials. Recently study suggested that semaglutide versus placebo reduced liver steatosis but not liver stiffness in subjects with non-alcoholic fatty liver disease assessed by MRI (69), which results of this study suggest that Semaglutide has no benefit in the improvement of fibrosis in NAFLD patients, which is consistent with previous studies. Collectively, significant improvement in biopsy‐confirmed liver histology with GLP‐1RAs treatment provides the most substantial evidence for the efficacy of GLP‐1RAs in the management of NASH, although the role of GLP‐1 RAs is still needed to be validated in large sample controlled trials with long‐term follow‐up.

This research has two important strengths. First, it is the first to evaluate the effect of GLP-1 RAs on IHA in patients with T2DM and NAFLD. Second, it provides further confirmation that GLP-1 RAs have a positive effect in these patients and improve IHA, liver enzymes, anthropometric variables, and metabolic indices.

However, the study also has some limitations that should be acknowledged. The first and foremost is the limited number of eligible studies and the small sample size of each individual study. Second, we integrated different RCTs that showed wide clinical heterogeneity in terms of duration, type of medication, drug dosages, and choice of the control group, we used a random-effects model and sensitivity analysis where possible to reduce the influence of these factors. Third, due to the invasive nature of liver biopsy, none of the included studies reported on liver biopsy as a diagnostic tool but relied on MRI to diagnose NAFLD, which is less accurate in demonstrating improvements in NASH.

In conclusion, treatment with an GLP-1RA improves IHA, SAT, VAT, inflammation markers, body composition, some glycemic indices and serum lipids to some extent. GLP-1RAs could be considered as a potential treatment strategy for patients with T2DM and NAFLD, if there are no contraindications. At present, there are few large prospective studies on GLP-1RAs in these patients. Further studies of this type are needed to understand the direct and indirect effects of GLP-1 RAs on the pathogenesis and prognosis of T2DM and NAFLD.

## Data Availability Statement

The original contributions presented in the study are included in the article/**Supplementary Material**. Further inquiries can be directed to the corresponding author.

## Authors Contributions

Conception and design of the study: SL. Screening of databases, extraction and analysis of the study data: YZ, JX, and DZ. Drafting of the initial manuscript: YZ and JX. Verification of the study methodology: YS, DZ, SC, and ZW. Revision of the manuscript: SL, YZ, and JX. All authors contributed to the manuscript and approved the submitted version.

## Funding

This study was supported by Sichuan Science and Technology Program (No.2017SYZF0002 and No.2021YFH0168).

## Conflict of Interest

The authors declare that the research was conducted in the absence of any commercial or financial relationships that could be construed as a potential conflict of interest.

## Publisher’s Note

All claims expressed in this article are solely those of the authors and do not necessarily represent those of their affiliated organizations, or those of the publisher, the editors and the reviewers. Any product that may be evaluated in this article, or claim that may be made by its manufacturer, is not guaranteed or endorsed by the publisher.
