# Supplementary material for: Efficacy and Safety of GLP-1 Receptor Agonists in Patients With Type 2 Diabetes Mellitus and Non-Alcoholic Fatty Liver Disease: A Systematic Review and Meta-Analysis
Source: Front Endocrinol (Lausanne). 2021 Dec 9;12:769069. doi: 10.3389/fendo.2021.769069 (PMC8696030; doi:10.3389/fendo.2021.769069)
Supplement: Supplementary file 1 [file DataSheet_1.docx]

Supplementary Material

# Appendix 1: Search strategy

**Appendix 1: Search strategy**

**Database: PubMed 10-07-21**

#1. Diabetes mellitus, type 2/

#2.(diabetes mellitus, noninsulin-dependent or diabetes mellitus, ketosis resistant or diabetes mellitus, stable or diabetes mellitus, type II or diabetes mellitus, maturity-onset or diabetes mellitus, maturity-onset or type 2 diabetes mellitus or type 2 diabetes).ti,ab

#3.#1 or #2

#4. Non-alcoholic Fatty Liver Disease/

#5. (nonalcoholic fatty liver disease or NAFLD or fatty liver, nonalcoholic or liver, nonalcoholic fatty or nonalcoholic steatohepatitis).ti,ab

#6 #4 or #5

#7. Glucagon-Like Peptide-1 Receptor/

#8.(Glucagon like peptide 1 receptor or GLP-1R receptor or GLP1R Protein or GLP receptor).ti,ab

#9.(liraglutide or lixisenatide or exenatide or dulaglutide or albiglutide or semaglutide).ti,ab

#10.#7 or #8 or #9

#11. #3 and #6 and #10

**Database: Web of Science 10-07-21**

11.3 and 6 and 10

10. 7 or 8 or 9

9.TS=Glucagon-Like Peptide-1 Receptor

8.TS=Glucagon like peptide 1 receptor or GLP-1R receptor or GLP1R Protein or GLP receptor

7.TS= liraglutide or lixisenatide or exenatide or dulaglutide or albiglutide or semaglutide

6.4 or 5

5.TS=nonalcoholic fatty liver disease or NAFLD or fatty liver, nonalcoholic or liver, nonalcoholic fatty or nonalcoholic steatohepatitis

4.TS=Non-alcoholic Fatty Liver Disease

3. 1 or 2

2.TS= diabetes mellitus, noninsulin-dependent or diabetes mellitus, ketosis resistant or diabetes mellitus, stable or diabetes mellitus, type II or diabetes mellitus, maturity-onset or diabetes mellitus, maturity-onset or type 2 diabetes mellitus or type 2 diabetes

1.TS= Diabetes mellitus, type 2

**Database: Embase 10-07-21**

1. Diabetes mellitus, type 2/

2.(diabetes mellitus, noninsulin-dependent or diabetes mellitus, ketosis resistant or diabetes mellitus, stable or diabetes mellitus, type II or diabetes mellitus, maturity-onset or diabetes mellitus, maturity-onset or type 2 diabetes mellitus or type 2 diabetes).ti,ab

3.1 or 2

4. Non-alcoholic Fatty Liver Disease/

5. (nonalcoholic fatty liver disease or NAFLD or fatty liver, nonalcoholic or liver, nonalcoholic fatty or nonalcoholic steatohepatitis).ti,ab

6 4 or 5

7. Glucagon-Like Peptide-1 Receptor/

8.(Glucagon like peptide 1 receptor or GLP-1R receptor or GLP1R Protein or GLP receptor).ti,ab

9.(liraglutide or lixisenatide or exenatide or dulaglutide or albiglutide or semaglutide).ti,ab

10.7 or 8 or 9

11. 3 and 6 and 10

**Database: Scopus 10-07-21**

#1.(“Diabetes mellitus, type 2 “or “diabetes mellitus, noninsulin-dependent” or (diabetes mellitus*) or(type 2 diabetes*)).ti,ab.

#2.(“Non-alcoholic Fatty Liver Disease “or NAFLD or” nonalcoholic fatty liver disease “or “fatty liver, nonalcoholic” or” liver, nonalcoholic fatty “or” nonalcoholic steatohepatitis”).ti,ab.

#3.(Glucagon-Like Peptide-1 Receptor or (GLP-1R*) or (liraglutide* or lixisenatide* or exenatide* or dulaglutide* or albiglutide* or semaglutide*)).ti,ab.

4.#1 and #2 and #3

**Database: Cochrane Central Register of Controlled Trials (CENTRAL) 10-07-21**

(“Diabetes mellitus, type 2”or “diabetes mellitus, noninsulin-dependent” or (diabetes mellitus*)or (type 2 diabetes*) in Title Abstract Keywords) AND(“Non-alcoholic Fatty Liver Disease “or NAFLD or” nonalcoholic fatty liver disease “or “fatty liver, nonalcoholic” or” liver, nonalcoholic fatty “or” nonalcoholic steatohepatitis” in Title Abstract Keywords) AND(Glucagon-Like Peptide-1 Receptor or (GLP-1R*) or (liraglutide* or lixisenatide* or exenatide* or dulaglutide* or albiglutide* or semaglutide*) in Title Abstract Keywords) AND("randomized controlled trial" OR "controlled clinical trial" OR randomized OR placebo OR "clinical trials" OR randomly OR trial NOT (animals NOT humans)) in Title Abstract Keyword.

**2.Appendix 2: Supplementary Figures**


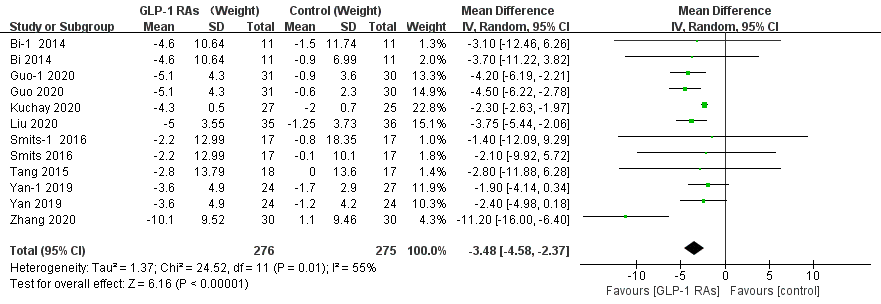


**Supplementary Figure 1** The forest graph of comparison of the mean change from baseline of weight between glucagon-like peptide-1 receptor agonists (GLP-1RAs) vs. control in patients with T2DM and NAFLD. SD, Standard deviation; CI, confidence interval; IV, inverse variance; Bi-1, concluded exenatide vs pioglitazone; Guo-1, concluded liraglutide vs insulin; Smits-1, concluded liraglutide vs placebo; Yan-1, concluded liraglutide vs sitagliptin.


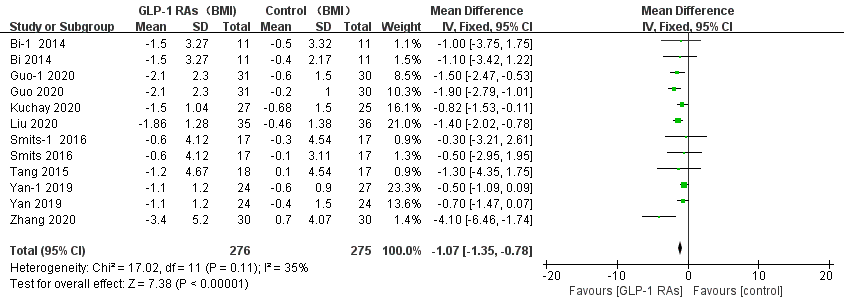


**Supplementary Figure 2** The forest graph of comparison of the mean change from baseline of body mass index between glucagon-like peptide-1 receptor agonists (GLP-1RAs) vs. control in patients with T2DM and NAFLD. SD, Standard deviation; CI, confidence interval; IV, inverse variance; body mass index (BMI); Bi-1, concluded exenatide vs pioglitazone; Guo-1, concluded liraglutide vs insulin; Smits-1, concluded liraglutide vs placebo; Yan-1, concluded liraglutide vs sitagliptin.

**
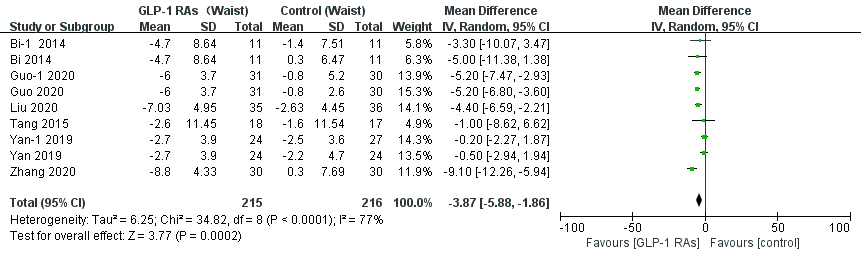
**

**Supplementary Figure 3** The forest graph of comparison of the mean change from baseline of waist circumference between glucagon-like peptide-1 receptor agonists (GLP-1RAs) vs. control in patients with T2DM and NAFLD. SD, Standard deviation; CI, confidence interval; IV, inverse variance; Bi-1, concluded exenatide vs pioglitazone; Guo-1, concluded liraglutide vs insulin; Yan-1, concluded liraglutide vs sitagliptin.

**
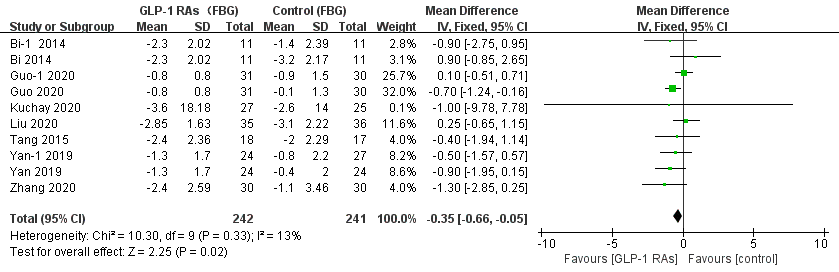
**

**Supplementary Figure 4** The forest graph of comparison of the mean change from baseline of fasting blood glucose between glucagon-like peptide-1 receptor agonists (GLP-1RAs) vs. control in patients with T2DM and NAFLD. SD, Standard deviation; CI, confidence interval; IV, inverse variance; fasting blood glucose (FBG); Bi-1, concluded exenatide vs pioglitazone; Guo-1, concluded liraglutide vs insulin; Yan-1, concluded liraglutide vs sitagliptin.

**
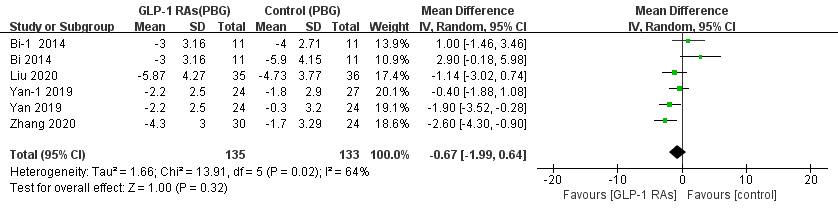
**

**Supplementary Figure 5** The forest graph of comparison of the mean change from baseline of postprandial blood glucose between glucagon-like peptide-1 receptor agonists (GLP-1RAs) vs. control in patients with T2DM and NAFLD. SD, Standard deviation; CI, confidence interval; IV, inverse variance; postprandial blood glucose (PBG); Bi-1, concluded exenatide vs pioglitazone ;Yan-1, concluded liraglutide vs sitagliptin.


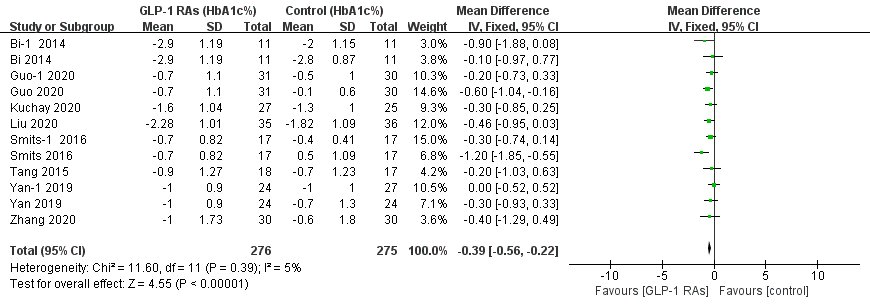


**Supplementary Figure 6** The forest graph of comparison of the mean change from baseline of the percent HbA1c between glucagon-like peptide-1 receptor agonists (GLP-1RAs) vs. control in patients with T2DM and NAFLD. SD, Standard deviation; CI, confidence interval; IV, inverse variance; Bi-1, concluded exenatide vs pioglitazone; Guo-1, concluded liraglutide vs insulin; Smits-1, concluded liraglutide vs placebo; Yan-1, concluded liraglutide vs sitagliptin.


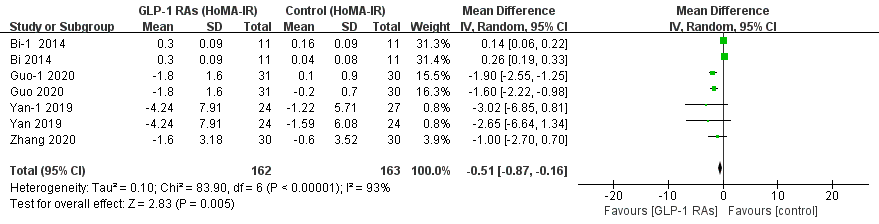


**Supplementary Figure 7** The forest graph of comparison of the mean change from baseline of HoMA-IR between glucagon-like peptide-1 receptor agonists (GLP-1RAs) vs. control in patients with T2DM and NAFLD. SD, Standard deviation; CI, confidence interval; IV, inverse variance; Bi-1, concluded exenatide vs pioglitazone; Guo-1, concluded liraglutide vs insulin; Smits-1, concluded liraglutide vs placebo; Yan-1, concluded liraglutide vs sitagliptin.


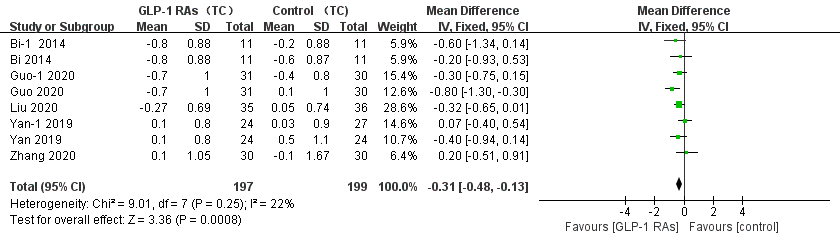


**Supplementary Figure 8** The forest graph of comparison of the mean change from baseline of total cholesterol between glucagon-like peptide-1 receptor agonists (GLP-1RAs) vs. control in patients with T2DM and NAFLD. SD, Standard deviation; CI, confidence interval; IV, inverse variance; total cholesterol (TC); Bi-1, concluded exenatide vs pioglitazone; Guo-1, concluded liraglutide vs insulin;Yan-1, concluded liraglutide vs sitagliptin.


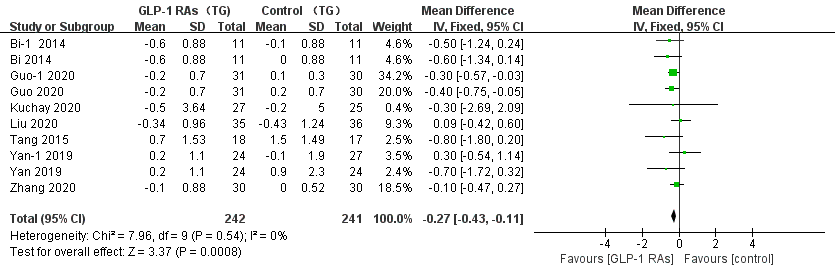


**Supplementary Figure 9** The forest graph of comparison of the mean change from baseline of triglycerides between glucagon-like peptide-1 receptor agonists (GLP-1RAs) vs. control in patients with T2DM and NAFLD. SD, Standard deviation; CI, confidence interval; IV, inverse variance; triglyceride (TG); Bi-1, concluded exenatide vs pioglitazone; Guo-1, concluded liraglutide vs insulin; Yan-1, concluded liraglutide vs sitagliptin.


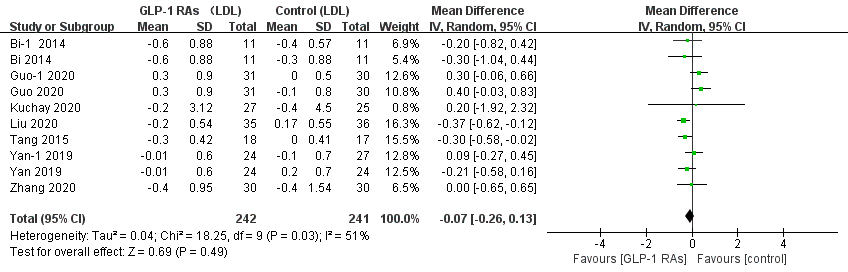


**Supplementary Figure 10** The forest graph of comparison of the mean change from baseline of low-density lipoprotein between glucagon-like peptide-1 receptor agonists (GLP-1RAs) vs. control in patients with T2DM and NAFLD. SD, Standard deviation; CI, confidence interval; IV, inverse variance; low-density lipoprotein (LDL); Bi-1, concluded exenatide vs pioglitazone; Guo-1, concluded liraglutide vs insulin; Yan-1, concluded liraglutide vs sitagliptin.


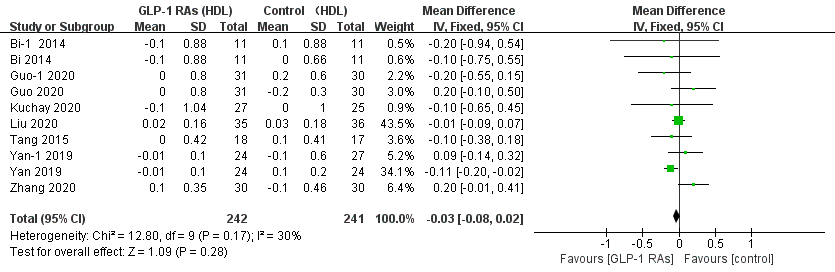


**Supplementary Figure 11** The forest graph of comparison of the mean change from baseline of high-density lipoprotein between glucagon-like peptide-1 receptor agonists (GLP-1RAs) vs. control in patients with T2DM and NAFLD. SD, Standard deviation; CI, confidence interval; IV, inverse variance; high-density lipoprotein (HDL); Bi-1, concluded exenatide vs pioglitazone; Guo-1, concluded liraglutide vs insulin; Yan-1, concluded liraglutide vs sitagliptin.


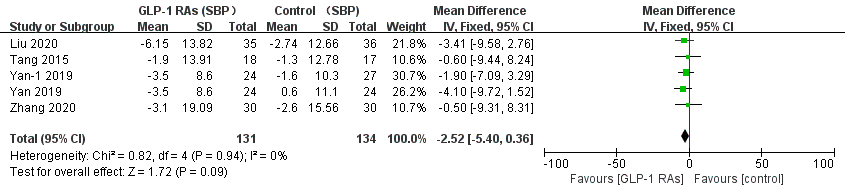


**Supplementary Figure 12** The forest graph of comparison of the mean change from baseline of systolic blood pressure between glucagon-like peptide-1 receptor agonists (GLP-1RAs) vs. control in patients with T2DM and NAFLD. SD, Standard deviation; CI, confidence interval; IV, inverse variance; systolic blood pressure (SBP); Yan-1, concluded liraglutide vs sitagliptin.


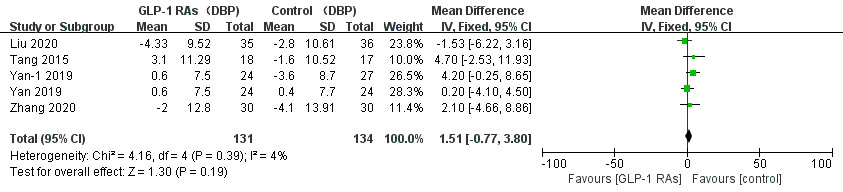


**Supplementary Figure 13** The forest graph of comparison of the mean change from baseline of diastolic blood pressure between glucagon-like peptide-1 receptor agonists (GLP-1RAs) vs. control in patients with T2DM and NAFLD. SD, Standard deviation; CI, confidence interval; IV, inverse variance; diastolic blood pressure (DBP); Yan-1, concluded liraglutide vs sitagliptin.
